# Cancer Mortality by Ethnicity in Colombia Between 2011 and 2022: A Population-Based Study

**DOI:** 10.3389/ijph.2025.1607975

**Published:** 2025-02-03

**Authors:** Maria Camila Urrea Suescun, Isabel C. Garcés-Palacio, Amr S. Soliman

**Affiliations:** ^1^ Department of Epidemiology and Biostatistics, CUNY Graduate School of Public Health and Health Policy, New York, NY, United States; ^2^ Epidemiology Group, School of Public Health, Universidad de Antioquia UdeA, Medellín, Colombia; ^3^ Department of Community Health and Social Medicine, City University of New York School of Medicine, New York, NY, United States

**Keywords:** afro-descendants, cancer mortality, Colombia, ethnic minority, indigenous people

## Abstract

**Objectives:**

To examine cancer mortality rates in Colombia by ethnic groups (Indigenous, Rom, Raizal, Afro-Colombian, and Mestizo) and assess trends from 2011 to 2022.

**Methods:**

National vital statistics from death certificates and the Colombian census data were used. Crude and direct age-standardized mortality rates were determined by ethnicity for the study period, by year, sex, and cancer type and Joinpoint analysis was conducted to examine trends.

**Results:**

Age-standardized cancer mortality of Mestizos (60.1 per 100,000 population) was lower than in Rom and Raizales (557.3 and 77.7 per 100,000), and higher than for Afro-Colombians and Indigenous (37.2 and 20.0 per 100,000). Indigenous people in Colombia had greater proportions of individuals under 45 dying of cancer than Mestizos (18.7% vs. 9.7%, p-value = <0.01). Compared to the Mestizo population, Raizales and Afro-Colombians experienced disproportionately higher age-standardized mortality rates due to prostate cancer (26.6/100,000 and 8.6/100,000 vs. 8.1/100,000), and for Raizales and Rom breast cancer (14.0/100,000 and 103.2/100,000 vs. 9.1/100,000).

**Conclusion:**

The disparities in cancer mortality in ethnic minorities in Colombia call for investigating cancer etiology and access to care among the Rom and the Raizal populations.

## Introduction

Research has shown that indigenous communities have poorer health outcomes, with a higher incidence, mortality, late-stage diagnosis, and less access to proper cancer treatment, arising from socioeconomic status as well as racial, cultural, and religious beliefs [1]. Studies conducted globally have identified barriers to cancer care such as lack of transportation for medical visits, shame, and general mistrust in the healthcare system [2, 3]. Similarly, the Romani ethnic group (also known as Roma in the UK) faces comparable barriers. Although, the research on this group is limited, some research has shown that they prioritize trusting relationships with health professionals but report a lack of access to interpreters during primary care visits leading to delays in cancer diagnosis, treatment, and care [4].

A well-established body of research also suggests that people of African descent in the western hemisphere have worse cancer outcomes overall [5–7]. Black people are more likely to be diagnosed with advanced stages of various cancers, including prostate, colorectal, multiple myeloma, pancreatic, breast, and lung cancers [8–10]. These disparities are likely due to lifestyle and environmental factors that disproportionately affect Black communities, as well as unequal access to high-quality treatment [6, 11]. Furthermore, studies in high-income countries and Latin America show that Indigenous and Black women have a higher risk of cervical cancer compared to other ethnicities [12–14], identifying high cost and lack of language access as major barriers to cervical cancer care in countries such as Peru and Colombia [15–19].

A Colombian-based study explored environmental risk factors concerning cancer in minorities, finding higher risks of neck masses in communities near hydrocarbon-contaminated water sources [20]. Additionally, statistics by the National Cancer Institute in Colombia suggest there is a higher prostate cancer burden in Afro-Colombian communities, based on the association between higher prostate cancer mortality rates and regions with larger Afro-Colombian populations [21]. A review by Moore et al. indicated that cervical cancer mortality risk is highest among Indigenous women from the Amazon region, and stomach cancer mortality is highest among Indigenous populations in the Cauca region [22]. However, these conclusions are based on cancer mortality reports by regions with higher proportions of ethnic minorities [22].

A projection for Colombia indicates that cancer incidence will increase by 45.8% between 2018 and 2030 and by 86.5% between 2018 and 2040 [23]. To address these concerns, the Ministry of Health established the 10-year Plan for Cancer Control in Colombia (2012–2021) to improve the quality of life and survival of cancer patients, but it did not address targeted approaches or research objectives focused on ethnic groups in Colombia [24, 25].

Colombia has four legally recognized ethnic groups: Indigenous, Afro-Colombian (including people of African descent, Black, Mulatto, and Palenqueros from San Basilio), Raizales from the archipelago of San Andrés and Providencia, and Rom (also known as Romani) [26]. As of the 2018 census, these groups represent 4.31%, 6.68%, 0.06%, and 0.01% of the Colombian population, respectively [27]. Although these groups may have worse health outcomes, little research has been done about them in the context of cancer, and no previous study has assessed cancer mortality rates for ethnic minorities in Colombia [28–30]. To address this research gap, this study aimed to analyze crude and age-standardized cancer mortality rates in Colombia by ethnic group over the entire study period on average and stratified by sex and common cancer types. As well as to assess yearly cancer mortality trends from 2011 to 2022 using Joinpoint regression, an established method for estimating changes in mortality rate trends [31].

## Methods

### Study Design and Data Source

This population-based study utilized mortality data that was consolidated, validated, processed, and disseminated by the National Administrative Department of Statistics (DANE) in Colombia. This information is obtained from death certificates processed digitally or physically by physicians, authorized medical personnel, and medical examiners [32]. The anonymized microdata for this study is available for public use and is accessible through the DANE website [32]. Additionally, publicly available Census data for years 2006 and 2018 were used for calculations requiring population size.

The DANE’s dedicated quality assurance team ensures data accuracy by generating revisions, addressing inconsistencies, and validating integrity, periodically updating vital statistics datasets. Additionally, studies have evaluated the quality of vital statistics and death certificates [33, 34]. One study found that as of 2016, less than 1% of deaths were not certified by physicians, with improvements in the quality of death certificates from 85.5% (1997) to 95.2% (2016) [34]. By analyzing all instances of cancer mortality for the entire Colombian population during the study period, selection bias was avoided. Furthermore, to ensure the use of the most current information, all datasets for this study were downloaded from DANE.gov on 19th June 2024 [35].

**FIGURE 1:**
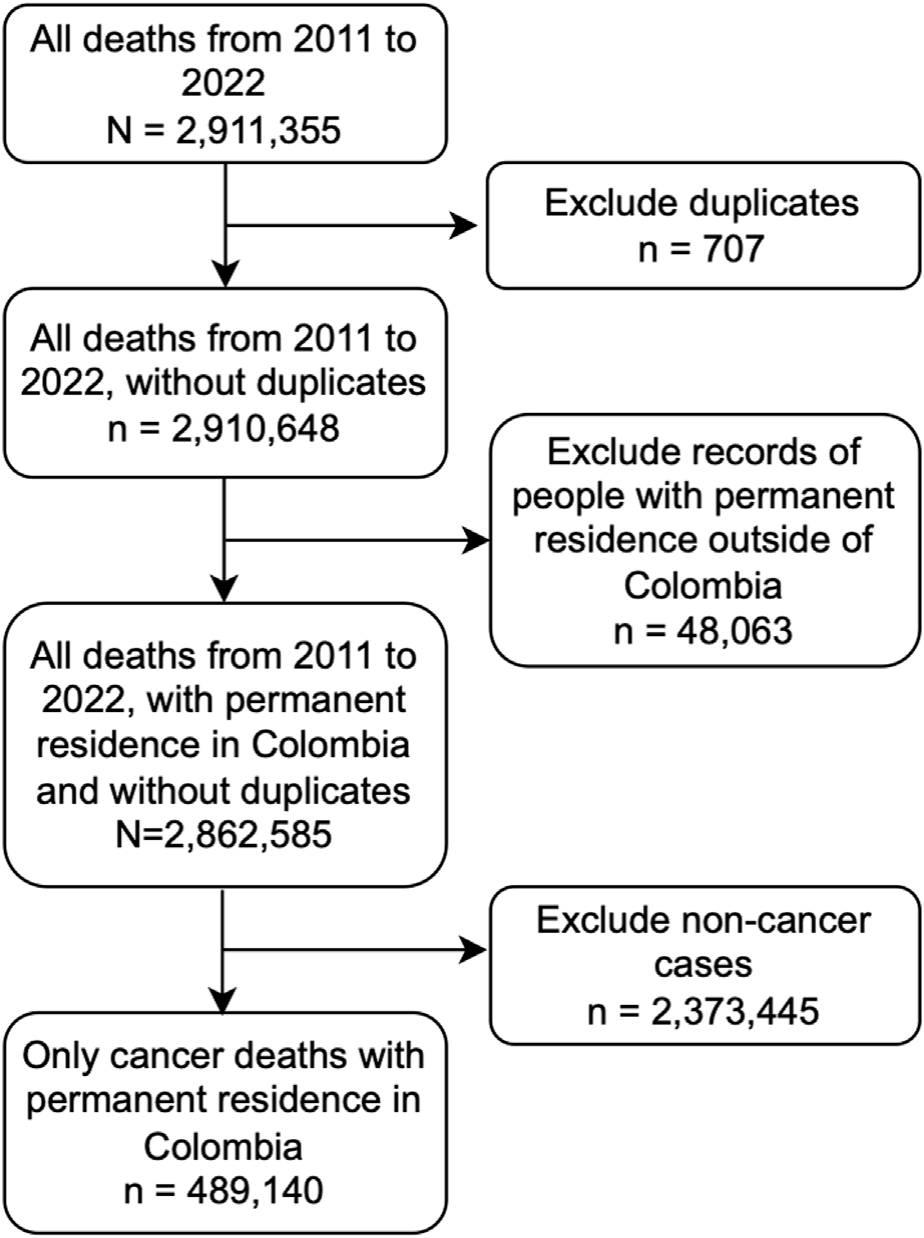
Data management of National Administrative Department of Statistics (DANE) vital statistics databases of 2011–2022. This graphic includes data cleaning steps and inclusion criteria, which resulted in a data set of 489,140 all cancer mortality data within the study period (Colombia, 2011–2022).

This study utilized non-fetal mortality data from 2011 through 2022. Each year has an individual data set which were reviewed to ensure all variables were equivalent across years before merging them to create one data set for the time period of interest. Although some variables were not present for some years datasets, no inconsistencies were observed across all equivalent variables in any of the years included in this analysis. Once merged, the total number of mortality cases between 2011 and 2022 added up to 2,911,355. Only individuals whose primary country of residence was Colombia and whose basic cause of death was cancer (defined as having ICD-10 codes between C00 and C97) were included, resulting in a dataset of 489,140 cases. (Figure 1).

### Measures

The dataset provided by the DANE included over 45 variables related to demographics (sex, age, marital status, ethnicity, educational level, social security system), residence location (place of residence, area of residence), death location (place of death, region of death), person certifying the death, causes of death (codified with ICD-10 standards), among others. For this study, the following variables were analyzed: age group, area of residence, basic cause of death, ethnicity, health insurance type, and marital status.

The variable “According to culture, people or physical traits, the deceased was or was recognized as” included Indigenous, Rom, Raizal, Afro-Colombian, and Palenquero, as well as a category for Mestizo, described as “none of the above.” All options were maintained in their original groups for this analysis, except for Palenquero, which was combined with Afro-Colombians due to the small sample size (n = 120) in this ethnic group. Palenqueros are Afro-Colombians who are direct descendants of enslaved Africans who established a town near Cartagena in 1619 after escaping slavery [36].

The variable age had 29 categories: nine categories for those under 4, 19 categories from 5 to 99 years old (grouped in 5-year intervals), a category for 100 years old and older, and unknown. For the purpose of this analysis, all individuals 11 months or younger were grouped into a single category. Additional categories were created with the following age ranges: 1–14 years, 15–44 years, 45–54 years, 55–64 years, 65–74 years, and 75 years or older. An additional age variable was created for age standardization in mortality rate calculations, including the categories: <15, 15–24, 25–34, 35–44, 45–54, 55–64, and 65+ years.

Marital status included the categories: not married and living two or more years with a domestic partner, not married and living less than 2 years with a domestic partner, separated or divorced, widowed, single, married, or unknown. These original values were then re-coded into “married or free union” (for those married or living with a domestic partner for any length of time), “separated, divorced, or widowed,” “single,” or “unknown.”

The variable sex included male, female, and unknown, which were unchanged for this analyses. Additionally, no changes were made to the variable “deceased’s habitual residence area,” which included the categories principal municipalities (urban areas), urban center (rural area with at least 20 houses, that do not belong to a principal municipality), and rural. Finally, the variable indicating the deceased’s social security program (type of health insurance), was included. The original options were contributory, special, exception (all three of which are private insurance options), subsidized (government sponsored), and uninsured. Subsidized and uninsured were retained, while all other categories were re-coded into contributory to indicate private insurance coverage.

### Statistical Analysis

Cancer mortality rates in Colombia between 2011 and 2022 were calculated and compared by ethnicity. Crude mortality rates were calculated for each ethnicity by year. Additionally, crude and standardized mortality rates were determined by ethnicity for the entire study period, as well as by cancer type and sex. Statistical analyses, namely, Pearson Chi-square tests and standardized residuals, were conducted using SPSS Statistics version 29. Crude mortality calculations were completed in Excel version 16.83, and standardized mortality calculations were completed using EpiDat version 4.7 – which uses confidence intervals for Pearson’s correlation [37]. The statistical software Joinpoint version 5.0.2 was used for the analysis of longitudinal trends using joinpoint regression models, to calculate annual percentage rate changes and statistically significant trends by ethnicity [38, 39]. Joinpoint regression is a collection of statistical models used to assess trends in disease burden over time, estimating the changing rule of mortality rates using the least square method to avoid non-objectivity [31].

The following formula, in which X represents each ethnic group, was used to calculate the average crude mortality rates during the study period:

$$Average\;crude\;cancer\;mortality\;rate\;\left(\%\right)=\frac{\;\#\;of\;cancer\;deaths\;in\;population\;X\;\left(between\;2011\;and\;2022\right)\;}{total\;population\;X\;\left(from\;the\;2018\;Census\right)\times \#\;of\;years\;in\;the\;study\;period\;\left(12\right)}\times 100,000$$

For the denominator of the yearly crude mortality estimates, the P_estimate_ (population estimate) formula was employed, where P_1_ represents the population of interest from the second-to-last census (2005 in our case), and P_2_ represents the population size from the latest census (2018). For 2018, actual census data was used. For subsequent years, average growth or decrease observed by year from the P_estimate_ formula were added.

$$P\;estimate=P_{1}+\frac{\#\;of\;months\;from\;P_{1}\;census\;to\;date\;of\;the\;estimate\;}{\#\;of\;months\;between\;census\;periods}\left(P_{2}-P_{1}\right)$$

The following formula was used to calculate the yearly crude mortality rates:

$$Yearly\;crude\;cancer\;mortality\;rate\;\left(\%\right)=\frac{\;\#\;of\;cancer\;deaths\;in\;population\;X\;on\;year\;Y\;}{P\;estimate\;of\;population\;X\;on\;year\;Y}\times 100,000$$

For site-specific crude mortality rates, the average crude cancer mortality rate formula was applied. It was crucial to consider that for sex-specific cancers (including cancers of the breast, prostate, cervix, ovaries, etc.), the denominator of the total population depended on the sex affected by the specific cancer site. However, for all ethnic groups except the total population, only the percentage of males and females was provided. Consequently, the exact number of males and females in each group was estimated based on the overall sex ratio for that ethnicity.

For age-standardized mortality calculations, the age groups <15, 15–24, 25–34, 35–44, 45–54, 55–64, and 65+ years were used. These rates were determined using EpiDat, employing a direct standardization method, using the World Health Organization standard populations as a reference.

The City University of New York (CUNY) Graduate School of Public Health and Health Policy and the University of Antioquia determined this study exempt from Institutional Review Board review, as it used publicly available data.

## Results

### Description of the Study Population

Between 1st January 2011, and 31st December 2022, there were 4,938 Indigenous people who died from cancer (1.0%), 257 Rom (0.1%), 353 Raizal from the Archipelago of San Andres and Providencia (0.1%), 17,492 Afro-Colombian (3.6%), 465,668 Mestizo or mixed (95.2%), and 482 cases with missing information for the ethnicity variable. Compared to the Mestizo population, the Indigenous and Rom populations had a higher proportion of all cancer deaths in children aged 1 to 14 (3.5% versus 1.1%). For the age category of 15–44, the highest proportions were for the Indigenous (14.9%) and Afro-Colombian populations (11.9%) compared to 8.6% in the Mestizo population. Deaths among the Indigenous population over 55 years of age were lower compared to the Mestizo population (69.3% vs. 79.4%), while in the Rom population, mortality was higher among those aged 75 and over (40.1% vs. 35.5%). The Raizal population presented very similar percentages of death in all age groups compared to the Mestizo population. For the Afro-Colombian population, the proportions were lower among those aged 65 and over (54.0% vs. 60.1%). These differences were statistically significant with p-values <0.01 (Table 1).

**TABLE 1:** Socio-demographic information of 488,708 cancer mortality cases between 2011 and 2022 by ethnic group (Colombia, 2011–2022).

Characteristics	Indigenous	Rom	Raizal from the archipelago of San Andres and Providencia	Afro-Colombian (including Palenquero from San Basilio)	Mestizo	Total	P-value*
	(n = 4,938)	(n = 257)	(n = 353)	(n = 17,492)	(n = 465,668)	(n = 488,708)	
	n (%)	n (%)	n (%)	n (%)	n (%)	n (%)	
**Age group** (missing = 14)							**<0.01**
0–11 months	14 (0.3)	0 (0.0)	0 (0.0)	11 (0.1)	206 (0.0)	231 (0.0)	
1–14 years	171 (3.5)	9 (3.5)	0 (0.0)	259 (1.5)	5,166 (1.1)	5,605 (1.1)	
15–44 years	734 (14.9)	19 (7.4)	34 (9.6)	2,075 (11.9)	40,278 (8.6)	43,140 (8.8)	
45–54 years	596 (12.1)	31 (12.1)	36 (10.2)	2,311 (13.2)	50,047 (10.7)	53,021 (10.8)	
55–64 years	815 (16.5)	39 (15.2)	69 (19.5)	3,401 (19.4)	89,983 (19.3)	94,307 (19.3)	
65–74 years	1,065 (21.6)	56 (21.8)	88 (24.9)	3,966 (22.7)	114,647 (24.6)	119,822 (24.5)	
75 years or older	1,542 (31.2)	103 (40.1)	126 (35.7)	5,468 (31.3)	165,332 (35.5)	172,571 (35.3)	
**Sex** (missing = 1)							**0.025**
Male	2,325 (47.1)	123 (47.9)	190 (53.8)	8,522 (48.7)	228,213 (49.0)	239,373 (49.0)	
Female	2,613 (52.9)	134 (52.1)	163 (46.2)	8,970 (51.3)	237,454 (51.0)	249,334 (51.0)	
**Marital status** (missing =46,706)							**<0.01**
Married or free union	2,505 (56.0)	122 (50.0)	163 (54.3)	8,043 (51.7)	223,670 (53.1)	234,503 (53.1)	
Separated, divorced, or widowed	974 (21.8)	83 (34.0)	67 (22.3)	3,672 (23.6)	115,000 (27.3)	119,796 (27.1)	
Single	991 (22.2)	39 (16.0)	70 (23.3)	3,844 (24.7)	82,791 (19.6)	87,735 (19.8)	
**Insurance type** (missing = 406)							**<0.01**
Contributory	343 (7.0)	189 (73.5)	213 (60.3)	6,409 (36.7)	248,698 (53.4)	255,852 (52.4)	
Subsidized	4,470 (90.6)	57 (22.2)	132 (37.4)	10,525 (60.2)	207,731 (44.6)	222,915 (45.6)	
Uninsured	119 (2.4)	11 (4.3)	8 (2.3)	545 (3.1)	8,876 (1.9)	9,559 (2.0)	
**Residence type** (missing = 215)							**<0.01**
Principal Municipalities	1,578 (32.0)	239 (93.0)	245 (69.4)	14,404 (82.4)	400,580 (86.1)	417,046 (85.4)	
Urban Centre	750 (15.2)	7 (2.7)	88 (24.9)	1,490 (8.5)	18,568 (4.0)	20,903 (4.3)	
Rural	2,603 (52.8)	11 (4.3)	20 (5.7)	1,583 (9.1)	46,337 (10.0)	50,554 (10.3)	

*The p-values provided describe the level of significance for the comparisons between the descriptive variables of interest and mortality within ethnic groups. This table does not show information on any other cancer mortality cases between 2011 and 2022 that were missing information on ethnicity (n = 432).

Marital status differences were significant across ethnic groups (p < 0.01). Most individuals who died of cancer were married or in a free union. The highest percentages were among Indigenous (56.0%), followed by Raizal (54.3%), Mestizo (53.1%), Afro-Colombian (51.7%), and Rom (50.0%). The percentage of separated, divorced, or widowed individuals was highest among Rom (34.0%) and lowest among Mestizos (27.3%). The proportion of single individuals who died of cancer was highest among Afro-Colombians (24.7%).

Insurance type distribution shows significant disparities across ethnic groups (p < 0.01). The majority of Rom (73.5%) and Raizal (60.3%) populations have contributory insurance, compared to Mestizo (53.4%), Afro-Colombian (36.7%), and Indigenous (7.0%). The subsidized insurance type was predominant among Indigenous (90.6%), followed by Afro-Colombians (60.2%) and Mestizos (44.6%). Uninsured percentages were low across all groups.

Residence type distribution shows a high percentage of Rom (93.0%), Mestizo (86.1%), Afro-Colombian (82.4%), and Raizal (69.4%) populations living in principal municipalities, whereas this proportion was lower among Indigenous (32.0%). A significant proportion of the Indigenous population (52.8%) resided in rural areas, with all other groups having percentages below 10% (p < 0.01) (Table 1).

### Average Crude and Standardized Average Mortality Rate by Sex and Ethnicity

**FIGURE 2:**
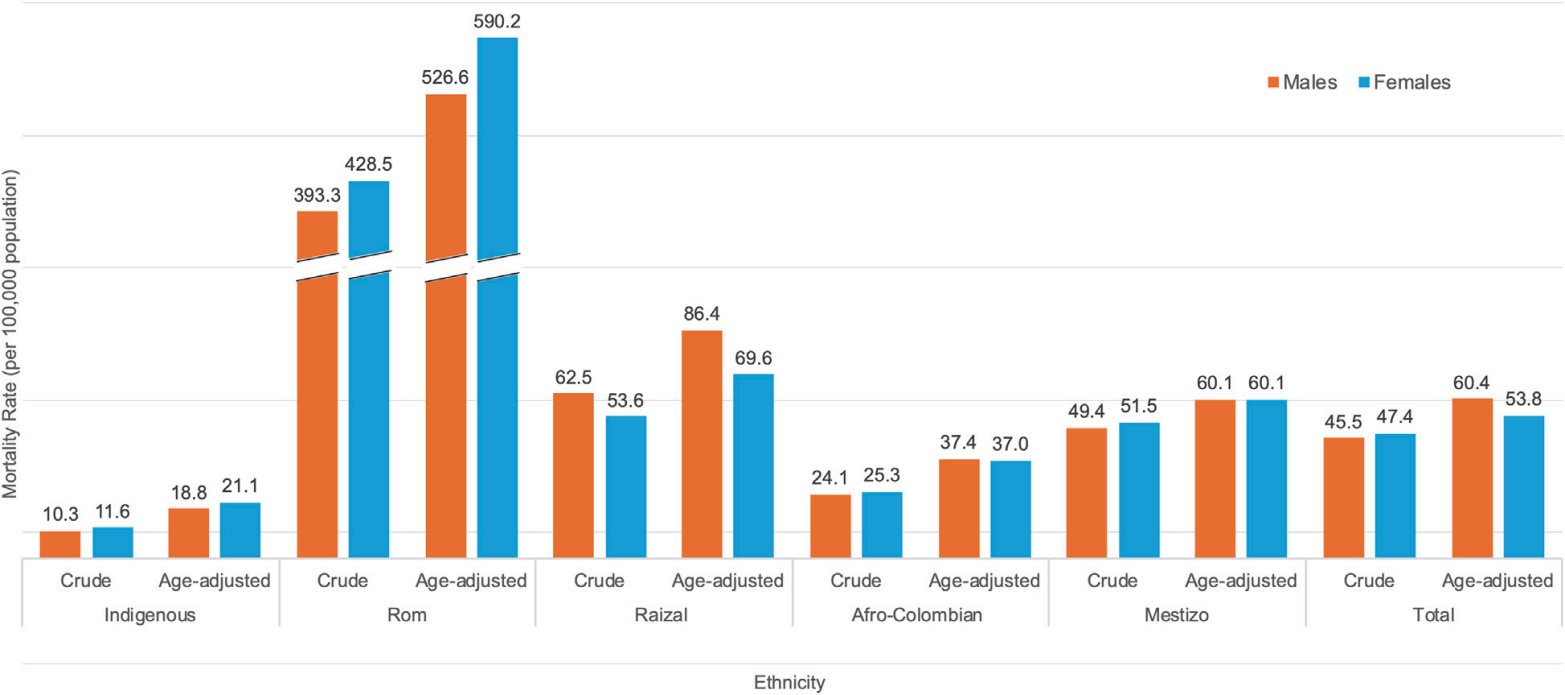
Age-standardized and crude cancer mortality rates (by sex) per 100,000 population, for each ethnicity in Colombia averaged between 2011 and 2022 (Colombia, 2011–2022).

As shown in Figure 2, for all ethnicities except Raizal, crude cancer mortality was higher in females than in males. Crude mortality rates for females compared to males were 3% higher among Mestizo, 5% higher among Afro-Colombian, 9% higher among Rom, 13% higher among Indigenous, and 14% lower among Raizal individuals. Conversely, after age-standardization, mortality rates were the same for Mestizo males and females. For Afro-Colombians, the proportion shifted from being higher in females to 1% higher in males. Similar to the crude rates, age-standardized mortality rates for females compared to males were 12% higher among Rom, 12% higher among Indigenous, and 19% lower among Raizal individuals.

The average crude and standardized cancer mortality rate per 100,000 people was highest for the Rom ethnicity (821.8 and 557.3), followed by Raizal (116.1 and 77.7), Mestizo (100.9 and 60.1), Afro-Colombian (49.4 and 37.2), and Indigenous (21.9 and 20.0). These rates contrast the overall crude and standardized cancer mortality rates for the total population, which were 93.0 and 57.1 per 100,000 population.

### Crude Mortality Rate by Year and Ethnicity

In the Indigenous group, the year with the lowest crude mortality rate was 2012, at 16.8 deaths per 100,000 population. On the other hand, the year with the highest mortality rate for this group was 2018, with 27.2 cancer deaths per 100,000. The annual percent change (APC) was estimated to be 3.27% during the study period; however, it did not show significant differences over time.

The differences by year showed greater variability in the Rom group, where cancer mortality was highest in earlier years. For instance, the crude cancer mortality rate was 1388.1 per 100,000 population in 2011 reaching the lowest mortality rate of 60.6 per 100,000 in 2022. Notably, this group experienced a significant decline (APC = −43.32%) in crude cancer mortality between 2019 and 2022.

The Raizal group experienced the highest crude mortality rate of 163.1 deaths per 100,000 population in 2017 and the lowest rate of 68.5 deaths per 100,000 in 2012. Similarly, the Afro-Colombian group’s lowest crude mortality rate from cancer was 37.9 deaths per 100,000 in 2012, while the highest was 53.2 deaths per 100,000 in 2022. Even though there were no significant changes in cancer mortality over time for the Raizal group, the Afro-Colombian group experienced a significant increase (APC = 6.52%) in cancer deaths between 2011 and 2015.

**FIGURE 3:**
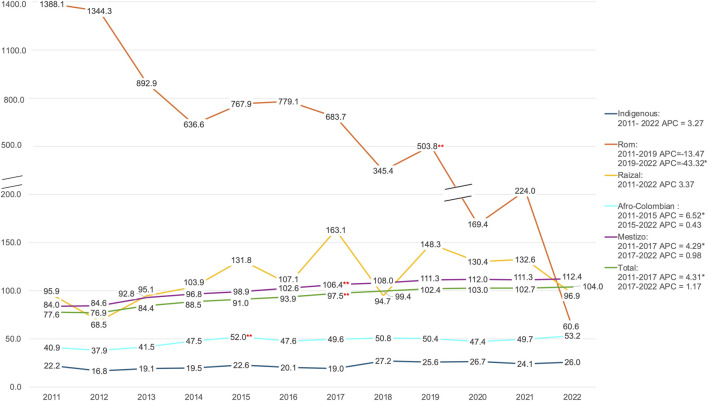
Yearly crude mortality rate in Colombia by ethnicity (per 100,000 population), between 2011 and 2022 (Colombia, 2011–2022). * Means the Annual Percent Change (APC) is statistically significant, ** signifies the presence of a joint point in the specified year in which the apparent change in trend is significant.

The Mestizo group experienced the lowest crude mortality rates in 2011 (84.0 per 100,000 population) and the highest in 2022 with 112.0 cancer deaths per 100,000. The APC between 2011 and 2017 was statistically significant at 4.29% (Figure 3).

### Age-Standardized Cancer Mortality Rate by Cancer Type and Ethnicity

**FIGURE 4:**
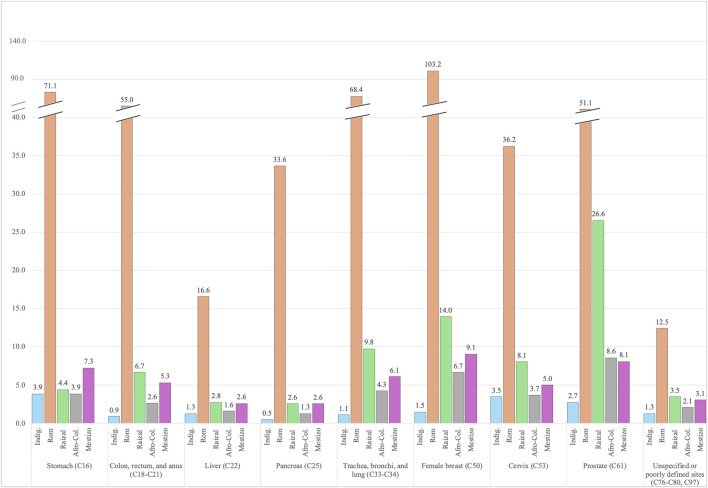
Standardized mortality rate (per 100,000 population) by ethnicity in Colombia, for the top 7 most common cancers among all ethnicities (Colombia, 2011–2022).

Among the seven most common types of cancer in each ethnic group, nine cancer sites were observed, all of which were significantly different (Supplementary Appendix A). The Rom ethnic group had the highest age-standardized mortality rate among all these cancers. They had mortality rates significantly higher than the Mestizo group for female breast cancer at 103.2 vs. 9.1, stomach cancer at 71.1 vs. 7.3, and cancers of the trachea, bronchi, and lungs at 68.4 vs. 6.1 per 100,000 population (Figure 4).

The Raizal group had the second highest standardized mortality rates in eight of the nine types of cancer, except for stomach cancer, where their mortality rate was lower than the Mestizo group at 4.4 vs. 7.3 per 100,000. The types of cancer with the highest standardized mortality rate per 100,000 in the Raizal community included prostate cancer (26.6 vs. 8.1), breast cancer for females (14.0 vs. 9.1), and cancers of the trachea, bronchi, and lungs (9.8 vs. 4.3).

Afro-Colombians had comparable standardized mortality rates per 100,000 population to the Mestizo group for their three most common types of cancer, including prostate cancer (8.5 vs. 8.1), breast cancer for females (6.7 vs. 9.1), and cancers of the trachea, bronchi, and lungs (4.3 vs. 4.3). On the other hand, the Indigenous group had lower standardized mortality rates when compared to the Mestizo group for all nine cancer types. The highest mortality rates per 100,000 for the Indigenous group were for cancers of the stomach (3.9 vs. 7.3), cervix (3.5 vs. 5.0), and prostate (2.7 vs. 8.1).

## Discussion

This study suggests that among all ethnic groups in Colombia, Indigenous people have the lowest cancer mortality rates overall, but conversely have the highest proportion of individuals under 45 years dying of cancer compared to any other ethnic group. Raizales and Afro-Colombians experience disproportionately higher mortality rates due to prostate cancer compared to the Mestizo population, while the Rom people exhibit the highest mortality rates for all cancer types in the nation. During the study period, an upward trend in cancer mortality was observed among most ethnicities (except the Rom), which was statistically significant among Afro-Colombians until 2015 and Mestizos until 2017.

Indigenous people had the lowest cancer mortality rates across the study period and among all cancer types analyzed in this study. This finding is consistent with the conclusions of a cross-sectional study conducted between 1985 and 2000 in Ecuador, which found that most cancers affecting a larger proportion of Indigenous people—such as stomach, cervical, and prostate cancers—had a significantly lower prevalence compared to non-Indigenous people [40]. In this study, we found that among the most common types of cancer leading to death among Indigenous people were cancers of the stomach, cervix, and prostate; however, both crude and standardized mortality rates for these cancers were lower than in the Mestizo population.

Notably, numerous studies—in high-income and Latin America countries—have found that Indigenous women disproportionately suffer from higher incidence of cervical cancer [13, 22, 41]. However, our findings suggest that although cervical cancer was the second most common type of cancer after stomach cancer in this population, it was still lower than in any other ethnic group. Lastly, this analysis suggests that, compared to other ethnic groups, Indigenous people have a higher proportion of deaths from infants as young as 1 month to adults up to 44 years of age. This is consistent with the findings of a study in Australia, which showed that Indigenous children had 1.37 times lower 5-year survival rates after a cancer diagnosis compared to non-Indigenous children [42]. At older ages, the proportion of Indigenous dying of cancer was much lower than in other ethnic groups.

Raizales had significantly higher mortality due to prostate cancer, as anticipated from a recent report by the National Cancer Institute of Colombia, which concluded that regions with larger Afro-Colombian populations had higher prostate cancer mortality [21]. Additional research has also shown that people of African descent in the Americas have disproportionately higher incidence and mortality rates due to prostate cancer, which has been attributed to a combination of genetic and environmental factors [6, 8, 9, 43]. Our findings support this, as age-standardized prostate cancer mortality was higher in the Raizal and Afro-Colombian groups as compared to the Mestizo population. Past research has also noted that Black females have a higher incidence and mortality due to breast cancer [44, 45]. Our findings partially support this, as age-standardized mortality rates were higher for Raizales but lower for other Afro-Colombians compared to the Mestizo population.

One potential explanation for why Raizales have higher cancer mortality rates than other Afro-Colombian groups is the impact of geographic location. Raizales are located in the Archipelago of San Andrés and Providencia – an island in the Caribbean. This community faces barriers to accessing care that Afro-Colombians on the mainland do not. One study highlights these barriers, noting that many Raizales avoid the healthcare system due to high out-of-pocket costs and a lack of access to specialized care [46]. While the government has recognized these barriers and is currently taking steps to develop resources on the island, a clear disparity still exists [47].

We would also like to highlight the significant decrease in the number of Afro-Colombians who self-identified as such in the 2018 Census (2,982,224) compared to the 2005 Census (4,311,757). The DANE, which is responsible for conducting the census, has identified errors in their sampling and will be taking measures to mitigate these mistakes in the future [48]. Therefore, although all estimates are based on the currently available data, there may be variability in the mortality rates if the Census data is incorrect.

Furthermore, our findings indicate that the Rom population exhibits the highest cancer mortality rates in Colombia, with breast cancer being the most common. A study in Spain found that the carrier frequency of the 185delAG BRCA1 mutation in Romani individuals could be several times higher than in the general population, putting them at a greater risk of breast cancer [49]. This could be a potential explanation to the findings in this study, which should be explored in future research. Studies in European Rom communities have also identified barriers to cancer care, including distrust of the medical system, fear of death, and language challenges [4, 50]. Given the distinct cultural context of Colombia’s Rom population – who established roots in South America in the mid-1800s and have since adopted a sedentary lifestyle – further research is needed to understand and address care disparities within this community and potentially develop public health strategies to improve their cancer outcomes [51].

According to census data, the Rom population decreased by 45.5%, from 4,857 to 2,649, between 2005 and 2018 [52]. This significant decrease has been speculated to have resulted from people not recognized as Rom, self-identifying as such in the 2005 Census, and better accuracy in the identification of Rom population due to the involvement of Rom community members who aided in collecting census data in 2018 [52]. Such disparities in population size may have influenced the calculated mortality rate estimates. Additionally, there was upward trend in cancer mortality for all ethnicities except for the Rom during the study period.

Similar studies have been conducted in other South American countries; however, this is the first study of its kind in Colombia, and the first to analyze cancer mortality in the Rom ethnic group within the country. The large sample size and the multiple years included in this study, makes these findings very valuable. Less than 0.01% of all mortality cases had missing data for the ethnicity variable, and the distribution of demographic characteristics was comparable among the cases with missing information and the Mestizo population, therefore, the majority of cases with missing ethnicity information were likely part of the Mestizo population. Notably, vital statistics in Colombia are a great source of mortality data. Given that this is a population study based on a robust data source, selection bias was not present, making these results generalizable to all the groups analyzed in the study.

It is important to highlight some of the limitations, inconsistencies in census data particularly changes in population size in the Rom community, and sampling issues in Afro-Colombian communities, may have influenced the significance of these findings. Future studies should perform these calculations again with more up-to-date population sizes. This study could not assess education attainment due to inconsistent data in the Vital Statistics datasets for this variable. Education could be a factor influencing cancer mortality and should be further analyzed. Additionally, all sex-specific calculations are estimates, as the Colombian census only provides overall sex percentages for each ethnicity.

In conclusion, this first-of-its-kind population-based study examined cancer mortality across different ethnicities in Colombia, revealing that Indigenous people have the lowest mortality rates, while the Rom population has the highest, particularly for breast cancer. The study observed an upward trend in cancer mortality for Mestizo and Afro-Colombians in earlier years of the study period. Raizales and Afro-Colombians faced higher mortality rates from prostate cancer, potentially due to genetics and barriers in healthcare access, especially for Raizales. Future studies should also focus on genetic variabilities within the Rom community that may put them at a higher risk of cancer, while also assessing the unique characteristics of this population that distinguish them from other Rom communities around the world. Finally, health services and access to care studies should focus on the barriers preventing Raizales from accessing proper cancer care, and how these may differ from the challenges faced by other Afro-Colombian communities.
