# Supplementary material for: Cancer Mortality by Ethnicity in Colombia Between 2011 and 2022: A Population-Based Study
Source: Int J Public Health. 2025 Feb 3;70:1607975. doi: 10.3389/ijph.2025.1607975 (PMC11830512; doi:10.3389/ijph.2025.1607975)
Supplement: Supplementary file 1 [file Table1.docx]

**Supplemental Materials**

**Supplementary Appendix A** Crude and age-standardized mortality rates (per 100,000 population) by sex and cancer type for all ethnic groups in Colombia (Colombia, 2011-2022).

| **Cancer Type** | **Ethnicity** | **Crude Mortality Rate** | **Age- Standardized Mortality Rate** | **95%** | |
| --- | --- | --- | --- | --- | --- |
|  |  | **(per 100,000)** | **(per 100,000)** | **Confidence Interval** | |
| **Malignant tumor of the stomach (C16)** | Indigenous | 4.3* | 3.9 | 3.6 | 4.1 |
|  | Rom | 108.7 | 71.1 | 49.1 | 104.0 |
|  | Raizal | 6.6* | 4.4 | 2.7 | 7.4 |
|  | Afro-Colombian | 5.2* | 3.9 | 3.7 | 4.0 |
|  | Mestizo | 12.5 | 7.3 | 7.2 | 7.3 |
|  | Total | 11.5 | 6.9 | 6.8 | 6.9 |
| **Malignant tumors of the colon, rectum, and anus (C18-C21)** | Indigenous | 1.0* | 0.9 | 0.8 | 1.0 |
|  | Rom | 86.3 | 55.0 | 36.2 | 84.8 |
|  | Raizal | 9.9 | 6.7 | 4.5 | 10.0 |
|  | Afro-Colombian | 3.5* | 2.6 | 2.5 | 2.8 |
|  | Mestizo | 9.2* | 5.3 | 5.3 | 5.4 |
|  | Total | 8.4 | 5.0 | 4.8 | 5.0 |
| **Malignant liver tumor (C22)** | Indigenous | 1.4* | 1.3 | 1.1 | 1.4 |
|  | Rom | 25.6 | 16.6 | 7.1 | 38.9 |
|  | Raizal | 4.3 | 2.8 | 1.4 | 5.3 |
|  | Afro-Colombian | 2.2 | 1.6 | 1.4 | 1.7 |
|  | Mestizo | 4.6* | 2.6 | 2.5 | 2.7 |
|  | Total | 4.3 | 2.5 | 2.4 | 2.5 |
| **Malignant tumors of the gallbladder and other parts of the bile ducts (C23-C24)** | Indigenous | 0.7* | 0.7 | 0.6 | 0.8 |
|  | Rom | 35.2 | 22.3 | 11.2 | 45.8 |
|  | Raizal | 2.0 | 1.2 | 0.5 | 3.4 |
|  | Afro-Colombian | 0.9* | 0.6 | 0.5 | 0.7 |
|  | Mestizo | 2.5* | 1.4 | 1.3 | 1.4 |
|  | Total | 2.3 | 1.3 | 1.3 | 1.4 |
| **Malignant tumor of the pancreas (C25)** | Indigenous | 0.6* | 0.5 | 0.4 | 0.6 |
|  | Rom | 51.2 | 33.6 | 19.1 | 60.0 |
|  | Raizal | 3.9 | 2.6 | 1.3 | 5.2 |
|  | Afro-Colombian | 1.8* | 1.3 | 1.2 | 1.4 |
|  | Mestizo | 4.6* | 2.6 | 2.5 | 2.6 |
|  | Total | 4.2 | 2.4 | 2.3 | 2.4 |
| **Malignant tumors of the trachea, bronchi, and lung (C33-C34)** | Indigenous | 1.2* | 1.1 | 1.0 | 1.2 |
|  | Rom | 105.5 | 68.4 | 47.0 | 100.7 |
|  | Raizal | 14.8 | 9.8 | 7.1 | 13.5 |
|  | Afro-Colombian | 5.9* | 4.3 | 4.0 | 4.4 |
|  | Mestizo | 11.0 | 6.1 | 6.0 | 6.2 |
|  | Total | 10.1 | 5.8 | 5.8 | 5.9 |
| **Malignant tumor of the female breast (C50)** | Indigenous | 1.7* | 1.5 | 1.3 | 1.7 |
|  | Rom | 164.8 | 103.0 | 66.6 | 162.4 |
|  | Raizal | 21.0 | 14.0 | 9.6 | 20.6 |
|  | Afro-Colombian | 9.0* | 6.7 | 6.3 | 7.0 |
|  | Mestizo | 15.2* | 9.1 | 9.0 | 9.1 |
|  | Total | 13.9 | 8.2 | 8.1 | 8.2 |
| **Malignant tumor of the cervix (C53)** | Indigenous | 3.8* | 3.5 | 3.2 | 3.8 |
|  | Rom | 52.7* | 36.2 | 15.5 | 83.6 |
|  | Raizal | 12.1 | 8.1 | 4.8 | 13.6 |
|  | Afro-Colombian | 4.9* | 3.7 | 3.4 | 3.9 |
|  | Mestizo | 8.0* | 5.0 | 4.9 | 5.0 |
|  | Total | 7.5 | 4.6 | 4.5 | 4.7 |
| **Malignant tumor of the body of the uterus (C54)** | Indigenous | 0.2* | 0.1 | 0.1 | 0.3 |
|  | Rom | 19.8 | 12.5 | 2.5 | 52.7 |
|  | Raizal | 5.1* | 3.3 | 1.4 | 7.8 |
|  | Afro-Colombian | 0.7 | 0.5 | 0.4 | 0.6 |
|  | Mestizo | 1.6 | 0.9 | 0.9 | 0.9 |
|  | Total | 1.4 | 0.8 | 0.7 | 0.8 |
| **Malignant tumor of the ovary and other annexes (C56, C570-C574)** | Indigenous | 1.0 | 0.9 | 0.7 | 1.1 |
|  | Rom | 26.4 | 16.5 | 4.5 | 57.6 |
|  | Raizal | 3.8 | 2.4 | 0.9 | 6.7 |
|  | Afro-Colombian | 2.0* | 1.5 | 1.3 | 1.7 |
|  | Mestizo | 4.9* | 3.0 | 2.8 | 2.9 |
|  | Total | 4.5 | 2.6 | 2.6 | 2.6 |
| **Malignant tumor of the prostate (C61)** | Indigenous | 3.1 | 2.7 | 2.5 | 3.0 |
|  | Rom | 80.7 | 51.1 | 27.2 | 98.4 |
|  | Raizal | 40.1* | 26.6 | 20.2 | 35.2 |
|  | Afro-Colombian | 11.8* | 8.6 | 8.2 | 8.9 |
|  | Mestizo | 14.9* | 8.1 | 7.9 | 8.2 |
|  | Total | 14.0 | 8.6 | 8.5 | 8.7 |
| **Malignant tumors of the brain and other parts of the central nervous system (C70-C72)** | Indigenous | 0.5* | 0.5 | 0.4 | 0.6 |
|  | Rom | 32.0 | 29.0 | 12.7 | 59.1 |
|  | Raizal | 2.3 | 1.6 | 0.6 | 4.0 |
|  | Afro-Colombian | 1.2* | 1.0 | 0.9 | 1.1 |
|  | Mestizo | 3.1* | 2.2 | 2.1 | 2.2 |
|  | Total | 2.8 | 2.0 | 1.9 | 2.1 |
| **Malignant tumors of unspecified or poorly defined sites (C76-C80, C97)** | Indigenous | 1.5* | 1.3 | 1.2 | 1.5 |
|  | Rom | 19.2* | 12.5 | -22.3 | 47.2 |
|  | Raizal | 5.6 | 3.5 | 2.1 | 6.2 |
|  | Afro-Colombian | 2.8* | 2.1 | 2.0 | 2.3 |
|  | Mestizo | 5.3* | 3.1 | 3.0 | 3.1 |
|  | Total | 4.9 | 3.0 | 2.8 | 3.0 |
| **Lymphoid leukemia (C91)** | Indigenous | 0.9* | 0.9 | 0.8 | 1.0 |
|  | Rom | 12.8 | 12.6 | 3.0 | 37.3 |
|  | Raizal | 0.3* | 0.4 | 0.0 | 2.5 |
|  | Afro-Colombian | 0.8 | 0.8 | 0.7 | 0.9 |
|  | Mestizo | 1.8* | 1.7 | 1.6 | 1.7 |
|  | Total | 1.7 | 1.6 | 1.7 | 1.6 |
| **Total Female** | Indigenous | 23.1* | 21.1 | 20.3 | 22 |
|  | Rom | 883.7 | 590.2 | 492.2 | 709 |
|  | Raizal | 103.9 | 69.6 | 59.2 | 82 |
|  | Afro-Colombian | 49.1 | 37 | 36.3 | 37.8 |
|  | Mestizo | 100.5 | 60.1 | 59.8 | 60.2 |
|  | Total | 92.0 | 53.8 | 53.6 | 53.8 |
| **Total Male** | Indigenous | 20.7* | 18.8 | 18 | 19.6 |
|  | Rom | 763.92 | 526.61 | 434.1 | 639.4 |
|  | Raizal | 129.1 | 86.4 | 74.5 | 100.4 |
|  | Afro-Colombian | 49.7 | 37.4 | 36.5 | 38.2 |
|  | Mestizo | 101.3 | 60.1 | 59.8 | 60.3 |
|  | Total | 92.6 | 60.4 | 60.2 | 60.6 |
| **Overall** | Indigenous | 21.9 | 19.9 | 19.4 | 20.5 |
|  | Rom | 821.8 | 557.3 | 489.0 | 635.45 |
|  | Raizal | 116.1 | 77.7 | 69.8 | 86.7 |
|  | Afro-Colombian | 49.4 | 37.2 | 36.6 | 37.7 |
|  | Mestizo | 100.9 | 60.1 | 60.0 | 60.2 |
|  | Total | 93.0 | 57.1 | 56.9 | 57.2 |

* Signifies a statistically significant difference in the number of cases compared to other ethnic groups.
